# Mapping emergent public engagement in societal transitions: a scoping review

**DOI:** 10.1186/s13705-021-00330-4

**Published:** 2022-01-05

**Authors:** Alexandra Revez, Niall Dunphy, Clodagh Harris, Fionn Rogan, Edmond Byrne, Connor McGookin, Paul Bolger, Brian Ó Gallachóir, John Barry, Geraint Ellis, Barry O’Dwyer, Evan Boyle, Stephen Flood, James Glynn, Gerard Mullally

**Affiliations:** 1 Department of Sociology and Criminology, University College Cork, Cork, Ireland; 2 Cleaner Production Promotion Unit, University College Cork, Cork, Ireland; 3 Environmental Research Institute, University College Cork, Cork, Ireland; 4 Department of Government and Politics, University College Cork, Cork, Ireland; 5 Institute for Social Science in the 21st Century, University College Cork, Cork, Ireland; 6 MaREI Centre, Environmental Research Institute, University College Cork, Cork, Ireland; 7 School of Engineering and Architecture, University College Cork, Cork, Ireland; 8 School of History, Anthropology, Philosophy and Politics, Queen’s University Belfast, Belfast, UK; 9 School of Natural and Built Environment, Queen’s University Belfast, Belfast, UK; 10 Irish Climate Analysis and Research UnitS (ICARUS), Department of Geography, Maynooth University, Maynooth, Ireland; 11 Center on Global Energy Policy, School of International and Public Affairs, Columbia University, New York City, NY USA

**Keywords:** Societal transitions, Emergence, Public engagement, Participation, Ireland

## Abstract

**Background:**

Transition discourses are gaining prominence in efforts to imagine a future that adequately addresses the urgent need to establish low carbon and climate resilient pathways. Within these discourses the ‘public’ is seen as central to the creation and implementation of appropriate interventions. The role of public engagement in societal transformation while essential, is also complex and often poorly understood. The purpose of this paper is to enhance our understanding regarding public engagement and to address the often superficial and shallow policy discourse on this topic.

**Main text:**

The paper offers a review of evolving literature to map emergent public engagement in processes of transition and change. We adopt a pragmatic approach towards literature retrieval and analysis which enables a cross-disciplinary and cross-sectoral review. We use a scoping review process and the three spheres of transformation framework (designated as the practical, political and personal spheres) to explore trends within this complex research field. The review draws from literature from the last two decades in the Irish context and looks at emergence and evolving spaces of public engagement within various systems of change including energy, food, coastal management and flood adaptation, among others.

**Conclusions:**

The results highlight the siloed and fragmented way in which public engagement in transitions is carried and we propose a more cross-sectoral and cross-disciplinary approach which depends on bringing into dialogue often contrasting theories and perspectives. The paper also illustrates some shifting engagement approaches. For instance, nexus articles between the practical and political spheres suggest deeper forms of public engagement beyond aggregated consumer behaviour to align technological delivery with institutional and societal contexts. While most articles in the practical sphere draw largely on techno-economic insights this influence and cross-disciplinarity is likely to draw in further innovations. Nexus articles between the political and personal sphere are also drawing on shifting ideas of public engagement and largely stress the need to disrupt reductive notions of engagement and agency within our institutions. Many of these articles call attention to problems with top-down public engagement structures and in various ways show how they often undermine and marginalise different groups.

## Background

The looming threat that climate change poses to humanity and the planet calls for an acceleration of societal transitions toward a low-carbon and climate resilient future. Research and policy debates in this area widely acknowledge that, to succeed, transitions must be based on cross-disciplinarity, knowledge co-generation and public engagement approaches [1–3]. The recognition that the transition process is fundamentally a social issue has deepened interest in leveraging public engagement and citizenship to build new pathways toward more sustainable futures. In this context, more passive notions of public engagement such as consumer choice are being challenged by emergent conceptions of citizenship with far deeper social and political ramifications [4, 5].

The relevance of engaging with these ideas is clear, but the process of grasping and supporting the role of public engagement toward societal transformation is complex. Fixing transition strategies around deeper forms of public engagement is made more difficult, because pinning-down definitions and roles for the public in sustainability transitions is much harder and more contested than commonly assumed [4]. Debates concerning sustainability transitions, for instance, continue to be stalled by unacknowledged tensions in relation to the potential benefits of participation, where there are winners and losers and numerous trade-offs, conflicts and challenges [6–8]. Renewable energy schemes such as large-scale windfarms, for example, are frequently proposed as an opportunity for collective growth and sustainability; yet these developments are often contested by local communities, as leading to significant devaluation of valued landscapes, livelihoods and homes [9, 10].

Equally problematic is the need for transition approaches based on disruption and acceleration, which can run counter to the acknowledged need for inclusiveness and reflexivity [11]. As we seek to open and widen networks and spaces for societal engagement we must address this tension between accelerating change, promoting disruption and ensuring societal cohesion and wellbeing [12].

Thus, trying to hold together a common vision of the future in the face of climate change which incorporate these tensions and promotes cogent and salient relationships for different groups, individuals and communities is both vital and very challenging [13, 14]. Emergent manifestations of inclusion, empowerment and participation relative to such complex change processes and systems will inevitably shape some forms of public engagement and displace others. Narratives about transitions need to find novel ways to better acknowledge, account and address these tensions and the fact that all public engagement conceptions might bring their own limitations, and forms of exclusion [5, 15].

Widening the debate on climate change in a manner that addresses this complexity requires a form of transitions research aimed at improving the means of shaping and anticipating complex change outcomes, embracing uncertainty, and producing more reflexive, holistic and responsive ways of learning and influencing transition processes [16–18]. Directing this perspective at public participation and engagement, highlights the value of accounting for emergence, ‘new technologies of participation’ and anticipating how they transform and reproduce power relations, social networks and collective agency in society [19–21]. So far, there has been limited attention paid to exploring these tensions and understanding public engagement ideas through such a lens. While fruitful, most research follows a deficit model approach, whereby the public engagement problem is framed around either lack of knowledge, capacity or motivation. It assumes that disinterest or dissent is due to a deficit in public understanding, and largely dismisses alternative framings and held values, as well as core political and personal struggles framing such dynamics [22].

This paper seeks to offer a critical map and review of the state-of-the-art of societal transitions research that relocates public engagement within a broader context and recognises wider political, personal and technological conditions in the making of ‘publics’. The paper acknowledges that the role of public engagement in societal transformation while essential, is also complex and often poorly understood. The purpose of this paper is to enhance our understanding of public engagement and to address the often superficial and shallow policy discourse on this topic. We adopt a cross-sectoral approach to widen and consolidate fragmented work in this field. Multi-sector approaches, which look across various systems, are an underutilized opportunity to consider more widely the effects and role of different actors in processes of change [3]. Recognizing the value of grasping wider interactions and public interface with societal transitions, we seek to offer an assessment of literature from the first two decades of the twenty-first century in Ireland. The review offers a map of emergent public engagement and explores how such interactions are shaped, challenged and stabilized within various systems of change including energy, food, coastal management and flood adaptation, among others. Thus, we strive to promote cross-disciplinary insights in this field, as well as contribute to a growing body of work on methods for mapping participation, which seek to enhance learning and reflexivity, by drawing together and making visible the complexity of techniques and processes that make-up this space [23].

The ‘mapping’ activity involves identifying Irish literature addressing public engagement and participatory approaches to societal transitions using a scoping review methodology and presenting ideas adopting a simple structure that positions different arguments within the three distinct spheres of ‘transformation’, designated as the practical, political and personal spheres [7]. The mapping approach alongside the three-spheres of transformation framework as an organising structure can be used as a tool to identify and compare how different public engagement interactions are arising, why they are arising and where they are arising [24].

### Sustainability transitions in Ireland

Transitioning to a low carbon and climate resilient pathway has become part of contemporary policy and research discourse. The urgency of this task is made clear in the recent Special Report on Global Warming of 1.5 °C (SR1.5) by the Intergovernmental Panel on Climate Change (IPCC) by forewarning policy makers that limiting global warming to 1.5 °C target is crucial to avoid catastrophic consequences [24]. The IPCC report also stresses that reaching this target will require “rapid and far-reaching” transitions in land, energy, industry, buildings, transport, and cities.

Yet in Ireland, as in many other nations, the notion of long-term exposure to climate change has been difficult to reconcile with shorter term political cycles and policy targets [25]. Thus, the social and political implications of living with climate change, which require considerable societal disruption and transformation has been diluted by agendas of change that still nurture ideological and institutional links to existing carbon economy regimes. Agendas based on inclusivity have also struggled to counter existing (ideological) top down centralised structures which facilitate and further institutionalize heightened wealth concentration and inequality, as well as centralization of planning and operation.

However, this situation is changing with new ambitious targets that seek to overcome Ireland’s status as a ‘laggard’ country relative to implementation of climate change policies [26]. Ireland has undertaken a multi-stakeholder initiative, the National Dialogue on Climate Action with the objective of raising awareness, engagement and mobilise action (locally, regionally and nationally) in relation to the challenges presented by climate change [27]. Four Climate Action Regional Offices involving all 31 Local Authorities in Ireland, have also been established to drive climate action, and support public engagement at regional and local levels [28]. More recently, the Irish government published the Climate Action and Low Carbon Development (Amendment) Act 2021 [29] which increased near term (2030) and long term (2050) mitigation ambition to a 51% reduction in GHGs and net zero GHGs, respectively (essentially doubling the rate of greenhouse gas emission reductions over the period 2020–2030). This Act also instituted the use of five yearly carbon budgets and cited “the requirement for a just transition to a climate neutral economy”.

Ideas about public engagement abound in new policy and legislative documents, this is exemplified by the acknowledged need of “bringing communities with us” as set out in the Programme for Government ‘Our Shared Future’[30]. EirGrid plc (the state-owned agency that manages and operates the transmission grid) published a recent report ‘Shaping our Electricity Future’, where it outlines a series of outputs based on community engagement [31]. It suggests ‘we listened’ and outlines direct interventions such as 0.5GW local renewable energy microgeneration ambition as a result of this engagement process. Yet, it has been noted that while transition policy and research with a public engagement focus is gaining momentum in Ireland, there is limited detailed understanding of transition frameworks in a manner that allow us to accurately position different actors in relation to systems of change [32, 33].

There are some noticeable developments which have gained wide attention in Ireland and elsewhere. A key development is the promotion of deliberative processes such as Citizens’ Assemblies, a democratic innovation that has become a feature of the Irish political landscape and that has been used to address issues as diverse and eclectic as Ireland’s abortion laws, referendum campaigns, and how the country should become a leader in its response to the climate emergency [34]. The increased use, and public acceptance, of Citizens’ Assemblies within the Irish policy landscape has created space for showcasing innovations, leadership and for the sharing of personal testimony. At present such emerging innovations such as the Citizens’ Assembly are pursued to accelerate processes of change, and in the Irish context they are often seen as the means to overcome its laggard status and leapfrog into a leading position toward effective and transformative climate action.

Overall, these developments suggest a considerable reimagining of public engagement regarding climate action and sustainable transitions. In this context of change it becomes important to explore public engagement not as a fixed category but rather as emergent and continuously shaped and enacted [4].

## Emergence and public engagement: an overview

Public engagement is best understood as a fluid category which considers the many instances in which citizens, communities and individuals deliberate, participate, inform, collaborate, intervene or actively oppose issues that concern them [35]. The notion of emergence, as such, is helpful to consider potential new spaces of public engagement, and further stresses the problem of portraying and containing the concept into a fixed definition [36]. As an inherently ambiguous concept it refers to a collective idea for democracy which is directed at multiple and often conflicting entities depending on context [37]. Offering a singular definition for public engagement cannot resolve the intrinsic vagueness and plurality of evolving meaning attached to the concept (*ibid*.). Indeed the value of participation, and in particular, from a transitions perspective, lies with the fact that new and experimental collectives may emerge that reframe problems and solutions in new ways [36].

Thus, the term is useful as an umbrella concept that incorporates a wider range of processes, dynamics and interactions taking place at various scales. While it is an unstable concept, it is valuable to help explore the evolving vocabulary required to make sense of the role of different actors and stakeholders in processes of change [38]. Such approach draws from multiple relationships with various actors and considers different roles beyond conventional definitions which tend to prioritise public engagement and participation in reference to institutions of government [39]. Or as publics with predefined citizen characteristics acting and behaving in predictable and predetermined ways. This more conventional view limits our understanding of public engagement and often binds it to formal decision-making processes and institutions. Such limitations account for the predominance of narratives which conceive engagement in narrower framings tied to social acceptance of new technologies, consumer behaviour and service use [5, 40–42]. In search of a more refined lens to make sense of emergent ‘publics’ it becomes necessary to acknowledge that some of these engagements fall in and out of favour, others are fleeting and others may express resistance, ambivalence or indeed refusal to participate [36, 43].

Drawing on Chilvers and Pallet’s [22] concept of ‘publics’ as relative to, and continuously shaped by, context, experience, technology, knowledge breakthroughs, policies, and institutional settings, emergence relates to wider and interconnected spaces of participation within and across different regimes and systems. In proposing a more interconnected and interactive definition of public engagement the authors recognise the need for a new dialogue which is less concerned with ranking the merits of specific public engagement approaches, but rather considers how these connect, merge, collapse and shift within the evolving context of change and transition [5].

To help make sense of some of these emerging debates this review article traces the application of these discourses in Ireland across a range of multiple perspectives and conceptual frameworks using the three spheres of transformation to structure this exploration. Below we offer an introduction to the three-spheres framework and outline the benefits of such approach to help integrate and synthesize cross-sectoral and cross-disciplinary literature in this field.

## The three spheres of transformation framework

To sustain wider and deeper dialogues across disciplines and at grassroots level, we must acknowledge that societal transition agendas are complex and fragmented and often operate within highly specialised fields that are guided by specific orientations and worldviews. Some attention has been dedicated to making sense of this growing complexity; examples of such research include attempts to: bridge different analytical perspectives [1, 44]; analyse prevalent concepts [45]; develop typologies of dominant theories [2]; and determine research agendas for sustainability transitions [3]. Reviews with a specific focus on participation and public engagement include: mapping policy change for public engagement with energy infrastructure in the UK [23]; systematic review of public participation in the UK’s energy transition [5]; and a systematic conceptual review of energy democracy [46].

While we recognise important conceptual and theoretical value in various societal transitions approaches, we seek to transcend more narrow representations of public engagement by adopting the three spheres of transformation as a heuristic device to help integrate evolving work in this field [24]. This framework offers no specific orientation or theoretical approach relative to change but instead it provides a structure that helps bring together the various approaches and theories that make up this field [24]. This is useful as it assumes a more pragmatic stance to explore across multiple frameworks and consider different dimensions of social transformations in a manner that is largely complementary to other theories [7, 24].

**Fig. 1:**
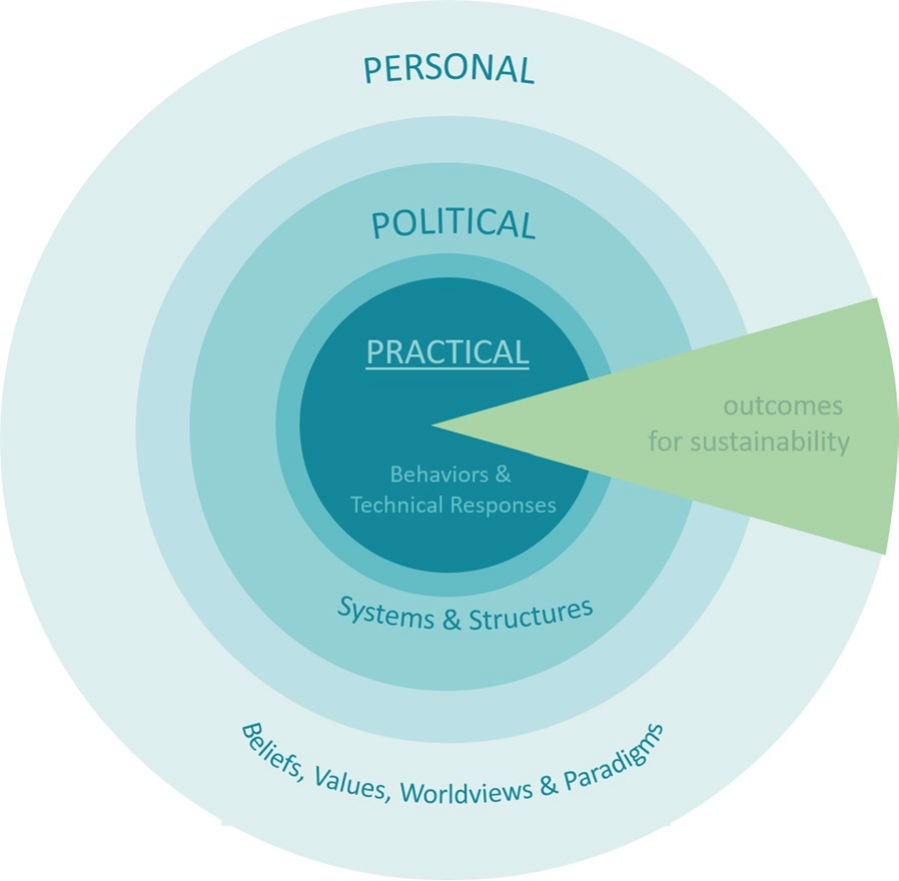
Three spheres of transformation framework (after Sharma [47] by O’Brien [24])

The framework was initially developed by Monica Sharma [47, 48] and subsequently refined by O’Brien and Sygna [7]. It has been adapted from empirical work in the field of integral theory and proposes a view of systems integrated and entangled with hierarchies stemming from collective and individual action. It recognizes the interdependence of behaviours, systems, culture and experiences, in how specific relationships are legitimised and prioritised. The notion of spheres is used to convey the idea of interrelated systems or fields of activity which constitute a larger whole [47], see Fig. 1. The three spheres, designated as the practical, political and personal sphere provide an organizing structure, which incorporate objective and subjective dimensions as well as collective and personal perspectives. We offer a description of each of the spheres below.

*Practical sphere* Is concerned with observable and quantifiable parameters. Insights and research in the practical sphere are largely conditioned by background and expertise in specialised fields and typically represents a more closed dynamic, commonly directed at these same experts. Knowledge and action focus on objective dimensions tied to technical responses to climate breakdown and unsustainability; it involves the introduction of new technologies, changes in management practices, cost–benefit based climate policies, information driven or rational choice models of behaviour change, and footprint measurements, among others. Climate mitigation and conservation measures have focused on this sphere of transformation [7]. In isolation, changes in this sphere can be problematic, with the emphasis on feasibility often leading to business-as-usual models of change. For example, Urry [49] argues that the replacement of petrol cars for electric vehicles (EVs) does not necessarily transform mobility systems. As such, the transition to EVs debate is an example of the limits of a narrowly technological ‘fuel focus’ which neglects examination of the wider systemic changes that are required.

*Political sphere* It concerns matters that are within the ‘public’ realm and involves citizen engagement and deliberations within wider societal debates. As such, it encompasses the political, economic, legal and social systems, and structures that define the range of possibilities and constraints framing societal and environmental transformations. This sphere considers matters which concern people collectively, but also issues that are deemed to require collaborative citizen inputs. A robust political sphere is considered vital for a working democratic system. It establishes and re-energises the parameters for involvement and participation through power and political influence and is a site of interaction and tension in response to social movements, lobbying and collective action campaigns in either support or against transformations [24]. Research in areas of socio-technical transitions and social practices tend to focus on this sphere giving particular attention to the political sphere as a key enabler (or inhibitor) of change [7].

*Personal sphere* This sphere constitutes the transformation of subjective individual and collective beliefs, values and worldviews [24]. This sphere has several distinguishing characteristics, namely, that it reflects on issues that are deemed of a more personal nature which includes experiences at individual or family level, or less formal exchange among friends and communities. It involves issues of identity, sense of self and, therefore, holds a strong representational influence in the way problems are framed, questions are asked, controversies are silenced, and solutions are prioritised. It represents the more subjective dimensions of knowledge and action [24]. This sphere holds influence in the development of new ‘action logics’, and paradigms and spanning boundaries and connections between different scales and perspectives [7, 24]. It is also important in the consideration of individual (as opposed to structural) agency within sustainability transitions. However, there are limitations in adopting highly abstract conceptions of change, and links to the political and practical sphere are essential for developing strategies that are salient and actionable.

Taken together the three spheres of transformation offer an insightful framework to situate existing research in societal transitions. It allows for a cross disciplinary understanding of different approaches to change in a manner which considers collective and individual action as well as objective and subjective knowledge and activities concerning change. These criteria are particularly useful for situating different pieces of research or activities. Critically, the way debates and practices may coalesce, merge or migrate from one sphere to another is also a revealing consideration, which points to the evolving, complex and heterogenous nature of societal transitions debates.

The positioning of the three spheres thus direct attention to the relationship between the practical, political and personal dimensions of transformation processes. The three spheres framework places the practical sphere at the centre, followed by the political sphere in the middle and the personal sphere as the outer layer. These nested levels of analysis are organised with the practical at the centre as representing more objective, tangible and measurable activities relative to a specific goal and the personal sphere as the outer layer representing more subjective measures and goals. Further to this the placing of the personal sphere as the outer layer also illustrates the pervasive influence that this sphere holds on the other layers [24].

## Steps towards mapping transitions literature in Ireland. A scoping review methodology.

The main approach to data collection utilised in this study was a scoping review methodology. Scoping reviews are utilised as a way to explore subjects, which are complex and cover wide areas of research [50]. The framework used to carry out the review was that proposed by Arksey and O’Malley [50] and added to this we incorporated a pre-defined analysis structure which makes use of the three spheres of transformation framework (discussed above). The process has included the development of a methodical and transparent process of literature search, screening and analysis, leading toward a structured presentation of results [51, 52].

### Data collection

The review followed a pre-established set of criteria. It was carried out in a staged way to ensure that search, retrieval and analysis was conducted in a rigorous manner. This included development of inclusion and exclusion criteria, identification of key bibliographical databases, retrieval and management of information, and review of key materials using a pre-defined review protocol.

#### Inclusion exclusion criteria

The literature search and retrieval criteria included sourcing available literature written in English between the dates of January 2000 and April 2020. Retrieval of literature focused on several materials and these included: published peer-reviewed articles, academic books and limited retrieval of grey literature. Grey literature was limited to published technical and research reports and white papers, and excluded doctoral dissertations, conference proceedings and other unpublished work.

Key words and areas of interest were used to create a more refined search within academic databases. Keywords were: (climate change OR sustainab*) AND (transition* OR transformation*) AND (Irish OR Ireland). The search was refined to keywords that appeared in the title or abstract. The databases searched were: Academic Search Complete (ASC/EBSCO host); Applied Social Sciences Index and Abstracts (ASSIA); JSTOR (multidisciplinary); Project Muse; ResearchGate (open source); Science Direct (multidisciplinary); Web of Science (Arts and Humanities); Irish EPA website; and Google Scholar.

#### Selection of relevant material

Search results in each database were sorted by relevance and key articles were identified manually from the results using a pre-defined protocol, which looked for papers with a focus on sustainability transitions in the Irish context. This was carried out by screening through titles and abstracts to identify further texts for elimination. For instance, a number or transition articles emerged in the areas of child development, Northern Ireland politics and migration which had no relevance to the review. Furthermore the screening involved a review of ‘borderline’ articles and reports which by and large appeared to have some adjacent connection to the theme or the Irish context, and required more careful consideration for either inclusion or exclusion [51]. To analyse the data a preliminary synthesis approach was adopted, followed by the subsequent structured analysis using the three spheres of transformation framework to structure, summarise, compare and recount the range of the materials retrieved.

### Synthesis of literature

The scoping review indicates that there is an emerging body of literature in Ireland contributing toward the societal transitions’ debates. Eighty-seven articles and reports were identified. Transition concepts while varied were for the most part, central themes within these items of literature. The use of theories of change associated with transitions were uneven, some articles looked in detail at ideas of societal transitions, while other papers adopted alternative lenses to contribute to debates and visions of low carbon and climate resilience. Equally, many papers were based on an in-depth focus of the Irish context (either at national, regional or local scales), while others offered a comparative analysis with European and International contexts.

The most common subject under which societal transitions are currently debated in Ireland are in relation to energy (see Table 1 below for a breakdown). Flooding, governance, marine research and food also feature as relevant categories. The category ‘other’ encompass a variety of themes that feature one or two papers and include articles looking at farming, water, transport, work practices, among others. There are limitations in the categorisation process, this was not always clear-cut, as there are intersecting themes between subject entry points into transitions and transformations research. We identified only four papers that address transition from a cross-sectoral perspective, and these were mainly found in the grey literature.

**Table 1:** Breakdown of number of research papers/reports by entry point

Energy	41
Flood	7
Governance	7
Food	6
Behaviour change	4
Marine/coastal research	4
Economy	3
Environmental activism	2
Media and communication	2
Other	12
Total	87

## Integrated overview of approaches to change in Ireland using the three spheres of transformation framework

In this section we offer a breakdown of the literature collected using the three spheres of transformation as a framework to situate existing transitions research and narratives linked to public engagement. We provide a breakdown of contributions that offer an explicit theorical focus linked to existing transitions literature and those who offer an explicit focus on public participation. Using the three spheres we identify dominant ideas around participation as well as some emerging trends. We recognise a degree of difficulty in placing different contributions within the three spheres framework as there are overlaps and blurred distinctions between these nested spheres. For this reason, we emphasise and make the distinction between contributions which we deem primarily centred around one specific sphere and those that are situated at the intersection with other spheres. Crucially emergence of innovative public engagement ideas appears more strongly within articles which are situated at the intersection of different spheres.

### Practical sphere: causes, parameters and technical solutions to sustainability

The breakdown in Table 2 below shows that most research from the practical sphere adopts a largely techno-economic centred approach, with fewer articles establishing stronger interlinks with the political and personal spheres.

**Table 2:** Practical sphere breakdown of societal transitions research using three spheres of transformation framework

Practical sphere centred contributions	Nexus with other spheres	Societal transitions theoretical focus	Public participation focus
20 Contributions	4 Contributions	8 Contributions	5 Contributions
Structural, technological and economic energy roadmaps and low carbon scenarios [54–64]; Historic review of electricity policy in Ireland [65]; Indicators of energy efficiency systems in the residential sector in Europe [66]; Bioeconomy and related supply chains in Ireland [67]; Windscape developments across Europe [68]; Citizen Investment in distributed energy generation technologies [69]; Behavioural change interventions for sustainable consumption [70, 71]; Climate scenarios for Ireland based on extremes from living memory [72]; Sustainability science and knowledge transfer using Integrated Coastal Zone Management innovations [73]	cVVP models for energy provision [74]; The role of HEIs in solar photovoltaic niche development [75]; A MLP perspective on marine wind energy and the North Sea Offshore Grid initiative [76]; ICCT and eating practices [77].	[59, 60, 66, 71, 74–77]	[69–71, 74, 77]

This pattern emphasises the ongoing challenge of reconciling technological and societal approaches to transition and stresses the need to continue to pursue approaches which seek to develop insights across this divide, even though interdisciplinarity is widely recognised as essential in sustainability research [53]. Energy is a leading entry point of discussion which centre around meeting renewable energy targets, energy security, energy consumption and models to accurately represent these.

A key underlying concern in contributions in the practical sphere relates to the development of long-term structural roadmaps and pathways toward low carbon energy use. Table 2 also shows that only five articles offer an explicit focus on public participation. However, there are insights that can be drawn from all these contributions regarding the way the ‘public’ is figured. Participation and public involvement in the practical sphere are frequently represented within a vertical, tiered structure and there are strong references to specific roles such as consumers, clients, prosumers and private investors.

Energy modelling papers, which feature strongly in this sphere, make use of aggregate population representations to speak of specific collectives. These aggregate population representations are used to extrapolate long term trends with regard energy consumption and demand, population growth, willingness to pay and economic impacts [56, 57, 60, 62, 64, 67, 68]. The Economic and Social Research Institute review of Irish energy policy [57] for instance, makes use of this form of collective representation to consider economic impacts of energy policy to the Irish consumer, while Devaney and Henchion [67] consider consumer acceptance and demand, in the development of the bioeconomy and related supply chains in Ireland.

A focus on behaviour change and social enterprise as a pathway toward community-led greening, is another example emerging from the literature. Rafferty [70] explores projects such as the Grow Dome in Dublin City and the O’Gonnelloe Exchange in County Clare. Both employ behaviour change and social enterprise approaches toward the development and maintenance of green physical and social infrastructure. Carragher et al. [88] outline multiple socio-economic drivers to enable behaviour change and sustainable consumption practices.

Finally, multi-stakeholder participation in energy modelling appears as an emerging frame within the practical sphere with a few contributions concerned with reconciling structural, technological and economic change with social and institutional perspectives; this is seen to foster greater energy efficiencies, enhance knowledge transfer and deepen understanding of uneven social outcomes regarding change processes [60, 63]. Stakeholder input was used to develop hybrid modelling scenarios with a stronger social dimension.

#### Practical sphere: nexus articles

The four articles with a demonstrable nexus between the practical sphere, and the political/personal spheres, express some concern toward the evolution and development of specific technologies. From community-based Virtual Power Plants (cVPPs) [74] offshore wind [76], information and communication technologies (ICT) in food futures [77], and photovoltaic (PV) energy in HEIs [75]. Issues highlighted include uncertainty, complexity, measuring impacts and benefits, financing, as well as network incompatibilities. All of these demonstrate difficulties with technological handover as it interfaces with institutional and societal issues. Public engagement appears in this context to situate these technologies into specific contexts and practices.

Community energy and the citizen as an energy supplier appear as an emergent concept that speaks of evolving visions for an ‘energy cooperative movement’. This features as a driver of energy transition, with a recent article by van Summeren et al. [74] examining cVPPs as a novel model for energy provision in Ireland, Belgium and the Netherlands. The energy cooperative vision suggests a much stronger role for Irish people and communities in energy generation, distribution and ownership. It is largely envisioned in this literature as way to mobilise greater financial participation within Ireland and promote acceptance of new energy technologies such as wind. Further insights from Van Summeren et al. [74] point to limitations in transposing the ‘community logic’ into an energy system bound by parameters set by incumbent energy producers. The study also highlights some institutional barriers and identifies this issue as requiring further research. It expresses concern for a shift of focus in the running of cVPPs from embracing a plurality of community values toward fixing decisions on a purely financial basis.

In exploring food futures Davies [77] makes use of participatory backcasting techniques to contest the dominance of technocentric visions of food into 2050 by engaging with relevant debates on eating practices and imbuing technology with social and political purpose. The author highlights the fact that at present uneven practices of food consumption are largely obscured and that inclusion of diverse communities is necessary to understand tensions between emerging technologies and citizen–consumer expectations.

Horan et al. [75] propose Living Labs as a user-centred approach which enhances ability to learn and experiment with new solar PV technologies. The authors identify several knowledge gains on performance and financing in using Living Labs insights. It proposes the university campus as a ‘microcosm of society’, whereby experimentation and demonstration would expedite local scale deployment of solar PV technologies (p.7). The paper proposes the higher education institutions as an intermediary to promote citizen and community participation in renewable energy generation, distribution, and energy efficiency.

### Political sphere: factors and structures that empower/disempower change

Contrary to the practical sphere, where few articles offered an explicit focus on participation, in the political sphere over 60% of papers and reports address public participation in some form. Energy and governance are main entry point of discussion. Table 3 offers a breakdown of contributions from the political sphere, which emerges as the dominant sphere in terms of contributions.

**Table 3:** Political sphere breakdown of societal transitions research using three spheres of transformation framework

Political sphere centred contributions	Nexus with other spheres	Societal transitions theoretical focus	Public participation focus
42 Contributions	13 Contributions	24 Contributions	34 Contributions
Transformative flood adaptation [78–81]; Flooding and changing social contracts [82]; Marine resource governance [83–85]; Environmental policy developments in Ireland [26, 33, 86–93]; Transformation of food production and consumption in Ireland [94, 95]; Societal-wide transitions to low-carbon sustainability in Ireland [32, 96]; Citizens’ Assembly on Climate Change [97, 98]; Community ownership and citizen investor models for energy transitions [99, 100]; Transdisciplinary methodologies towards low carbon energy transitions [101, 102]; Classification of behaviour change initiatives [103]; A partnership approach toward sustainable SDG based transformations [104]; Transport governance in Ireland [105]; MLP based review of telework in Ireland [106]; Climate justice pathways toward zero carbon emissions by 2050 [107]; Sustainability strategies in HEIs [108]; Sustainability transitions in European residential sector [109]; Econometric analyses to predict acceptance of different energy infrastructural schemes [110]; Analysis of energy poverty in Europe [111]; Employment vulnerability and a just transition in Ireland [112]; Public participation, and climate change adaptation [113]; Environmental crisis and the wider crisis of capitalism [114]; Energy transitions in Europe and impact on energy relations and energy security [115]; Public discourse on smart grid technologies based in Irish media narratives [116]	Bio-financialisation of Irish Water [117]; Community led innovation pathways to transition [118]; The nexus between food, energy and climate [119]; Environmental activism in Ireland [120]; The Transition movement, emergence and geographical spread [121]; Energy citizenship concepts [42]; Degrowth strategies [122]; Divestment and energy justice [123]; Intersectional analysis of community energy practices [124, 125]; Risk perceptions of smart-farming technologies and practices [126]; Transdisciplinary frames towards sustainability [127]; Community drivers toward decarbonization [128]	[32, 33, 42, 78–81, 84, 93, 96, 104, 106, 114, 116, 118, 122–125, 127–131]	[26, 32, 78, 80–82, 84, 85, 89, 93–95, 97, 98, 100–104, 110, 112, 113, 116, 118, 120, 121, 123–128]

A key underlying concern entails the development of innovative social and political democratic infrastructure to support and promote societal transition ideas. A number of these articles express the need for the development of more inclusive and reflexive knowledge creation and decision-making processes. In institutional terms this is envisioned as a means to address the need for promoting governing structures that can anticipate and respond to uncertainty in change processes and constructively consider the standpoint of emerging individuals and groups. The literature on governance highlights the need for deep institutional transformation to overcome the institutional trap often implicated in slow processes of change.

In this context, different modes of participation are explored as important, these include: participatory, multi-level and reflexive governance [32, 78–84, 86, 87, 89, 93, 95, 96, 104, 105, 112, 113, 117, 118, 126, 128, 132]; participatory methods [101, 102, 109, 113, 124], lobbying [86, 87, 105]; deliberative fora [26, 89, 97, 98, 101, 104]; community development and interventions [32, 93, 103, 109, 112, 121]; and ownership [42, 85, 100, 110, 118, 126].

In terms of citizenship, there are significant developments that have received wide attention in Ireland and internationally. Namely, the literature has grown to include recently established deliberative processes such as Citizens’ Assemblies, a democratic innovation that has become a feature of the Irish political landscape and that has been used to address diverse issues such as Ireland’s abortion laws, referendum campaigns, and more recently the climate emergency [34]. Novel and emergent forms of deliberative democratic fora such as the Citizens’ Assembly and the National Dialogue on Climate Action have promising applications on sustainable transitions and transformations research [27]. To date this includes scholarly work that has championed the development of deliberative democratic innovations, as well as specific analysis of the Citizens’ Assembly (2016–2018) work on climate action [97, 98].

Three specific ‘publics’ dominate this discussion, they are: conceptions of citizenship [42, 82, 88, 89, 93, 97, 98, 101, 113, 114, 116, 123]; stakeholder participation [32, 33, 78–81, 83–85, 87, 88, 92, 93, 96, 100, 104, 118, 128]; the participation/protection of vulnerable groups [93, 103, 107, 111, 118, 124, 132, 133].

Community development, as in the promotion of planned and organised processes through which individuals and communities can learn and take control of issues that affect them, was identified as an area of emerging importance. Moore [93] identifies the work by communities as a tool for transition, including the EU-led RESCoop toward the promotion and support of renewable energy cooperatives, community supported agricultural projects, the Tidy Towns community initiatives and other less formal community initiatives such as plastic free and zero waste initiatives. The establishment of the local Public Participation Networks (PPNs) by local councils across Ireland is also seen as a tool for transition at local community level [93]. Similarly, Ellis et al. [32] look at the growing number of promising community energy initiatives, which have been incentivised by the Sustainable Energy Authority of Ireland (SEAI). These are seen to open many possibilities for the emergence of relevant niche social innovations and practices that may result in wider regime changes. Other relevant initiatives include social enterprise interventions such as community energy cooperatives and community interest companies, which are suggested by Morrissey et al. [118] as community niche innovations which represent a credible means to address vulnerability and focus on social, environmental and economic multiple-bottom lines [134]. Walsh [100], however, offers evidence which indicates that community-initiated energy is still largely aspirational in Ireland with few practical projects and limited literature to learn from, to help define what is still a ‘fuzzy’ concept. Namely, what constitutes community energy and who benefits from this.

The literature we identified on stakeholder engagement conveys the need for the political system to undergo deep institutional transformation towards more inclusive forms of policy development and implementation with those directly invested or affected by specific policies [80]. In our review we found that the emergence of opposition groups such as Save Cork City, the Clontarf Residents Coalition, and the Skibbereen Flood Forum, objecting to local flood defense strategies, exemplify the growing discontent of flood impacted communities with state organisations such as the Office of Public Works^1^ (OPW). This literature shows that engagement with local stakeholders is often characterised by an overreliance on one-off consultation events, and highly tiered stakeholder engagements. Events, which are arguably set with the sole purpose of meeting EU Flood Directive requirements for public engagement, are tokenistic and counterproductive. Failure to recognize stakeholder inputs more widely in this instance has led to a significant and costly impasse in the development and implementation of much needed flood management solutions [80]. It has also led to mismatched expectations concerning the role that the state plays in providing protection and support to impacted communities, suggesting a critical need for a new ‘social contract’, which more clearly defines the role both the state and communities should play in new processes of climate adaptation [82].

Institutional reform with a focus on inclusive stakeholder involvement is also highlighted in the literature looking at marine governance [83–85]. Kelly et al. [84] flag the need for the creation of well anchored institutional spaces to drive transformation and mediate between different groups. Transformation is seen to be stalled as a result of uneven stakeholder influence and engagement, since efforts to accommodate more influential stakeholders may lead to a selection of incremental changes rather than paths that contest established systems and regimes [84]. Using a Transition Management approach, the authors propose the setup of ‘transition arena groups’ to oversee a transition agenda with predefined outreach and inclusive strategies. This suggests a more explicit role for intermediary actors and has been highlighted elsewhere as a promising strategy to accelerate change [3].

The involvement of ‘key stakeholders’ has historical roots in Ireland, namely, the development of social partnership structures that have been an established part of Irish political life [135]. While this was often hailed as a model of successful economic governance by policy makers across Europe, many have labelled it as ‘democratic corporatism’ and argue that instead of giving marginalised communities voice, the environment of partnership acted as a ‘straight jacket’ which served specific interests and significantly hindered processes of change [136, 137]. Particularly as it gave little scope for differences of opinion, sense of agency and held back groups on developing and maturing their self-organization skills. Irish social partnership arrangements struggled to cope with challenges from the global economic turmoil in 2008, and the partnership model has significantly weakened since. However, there is a legacy from this model, particularly in terms of uneven interactions between powerful and well organised/institutionalised stakeholders (Farmers’ associations and employers confederations for instance) and those at the periphery, which is still prevalent, with a number of articles noting that powerful stakeholders often dominate and unevenly influence agendas of change [84, 87].

Evolving notions of vulnerability in the context of transitions speak of different capacities to adapt, have a say and influence over changing circumstances. Vulnerability and transitions in the energy system in particular emerge as a relevant theme in the literature identified [111, 118, 124]. Ireland was one of the first countries in Europe to officially recognize energy poverty as a public policy issue, and policy continues to evolve in this area [111, 138]. Energy Action CLG established in the 1980’s, was Ireland’s first community focused energy project to address the problem of energy poverty in Dublin. The number of groups and organisations with an energy poverty remit has grown. This stems from a growing awareness and consensus over the necessity to understand how transitions disrupt the capacity of different groups and individuals to sustain their wellbeing and livelihoods [93, 101, 111, 112].

#### Political sphere: nexus articles

Energy entry points continue to dominate as a theme within nexus articles linked to the political sphere. Yet, while national and public policy development was a core theme more closely aligned with the political sphere those at the nexus with other spheres have a clearer concern with ‘democratizing energy transitions’. Energy justice and energy democracy feature as an important criterion to scrutinize and refine existing visions of change. These emerging debates speak of environmental justice issues more generally, looking at processes of dispossession, devaluation and exclusion along multiple scales and perspectives, particularly those of underrepresented publics, such as vulnerable groups, future generations and ‘frontline communities’ [42, 123]. Lennon et al. [42] offer a critique of current energy citizenship constructs and highlight the limiting consequences of conceiving citizens as solely economic actors participating in the public sphere through consumer led choices and highlight important private sphere considerations such as issues of gender, care and homemaking.

Other alternative active citizenship visions emphasise the potential that political activism, collective mobilisation and community-led innovations should have in promoting substantial reform and transformation by questioning overly prescriptive views of what counts as participation and who establishes the parameters for debate. For instance, the divestment movement is advanced by Healy and Barry [123] as a transformative and disruptive form of action. The divestment movement is seen to draw attention to key ethical, intergenerational, ecological and financial issues associated with ‘business as usual’ and technology-led models, and make imperative, timely transformative political action.

Disruption and protest emerge again in terms of opposition groups linked to the Right2Water campaign, which emerged in the context of grassroots attempts to reverse the controversial setting of water charges in Ireland in 2015. This was a period of economic recession reflecting the confluence of economic and environmental issues. This effort received strong support from the general public, and forced the government to backtrack its policies on the financing of water services [117]. It has also arguably made the government more wary of introducing other environmental charges. This form of mobilisation may suggest a shift in the manner people mobilise around environmental issues. A notable feature of activism in Ireland has been the place-based and highly localised character of most movements; a prominent example is the protest against the sitting of a natural gas processing plant in North Mayo [120]. However, issues like the Right2Water campaign, the global-led Extinction Rebellion movement and the Global Climate Strike movement led by student groups Fridays for Future Ireland, who have wider national and international links (albeit somewhat fringe) suggest changes in the way groups mobilise and protest.

### Personal sphere: discourses and paradigms on individual and collective beliefs, values and worldviews

As Table 4 below indicates this is the least leveraged sphere of transformations in existing research in Ireland, indicating a trend toward continued emphasis on technological innovation and institutional policy-driven frameworks. Furthermore, there is also no discernable theme or entry point that align these different articles. While energy was a dominant concern across the practical and political spheres it is not a dominant entry point in the personal sphere. Given the smaller number of personal contributions, the degree to which participation features in the literature is high. Contributions centered around the personal sphere focus on disentangling more subjective and peripheral aspects of change drawing from more peripheral views, grassroots or value-led perceptions and experiences of change.

**Table 4:** Personal sphere breakdown of societal transitions research using three spheres of transformation framework

Personal sphere centred contributions	Nexus with other spheres	Sustainability transitions theoretical focus	Public participation focus
6 Contributions		3 Contributions	5 Contributions
Climate change media communication [139, 140]; Storylines of post-carbon rural transitions [9]; The food sovereignty movement [141]; Community based flood risk management perspectives in Ireland [142]; Modernity, permanent liminality and recent transition experiences in Ireland [143]	As above, main nexus with political sphere	[140–143]	(9, 139–142)

Approaches to societal transitions are diverse and include conceptions of liminality, risk management, transition food movements, rural transitions and media representations of low carbon transitions.

From a food system perspective, Sage [141] highlights the potential of food narratives to develop stronger links of community solidarity, as a unique axis of change, and as a baseline for a healthy resilient future. Social movements, food citizenship, local community eating, and food growing practices are emphasised as unfolding and evolving practices. The Transition Movement (TM)¸ with roots in Kinsale in County Cork and now spread globally [141] is highlighted as a particularly prolific organisation, having become the fastest growing environmental movement in the global north [141]. TM has proven to be highly relevant to local communities, particularly those already engaged and looking for models of action to work within smaller scale, localised areas [121]. However, this wide exploration of a very broad range of food initiatives and groups signals the emergence of potentially limiting community practices. Sage finds that often present-day food transition visions assume an ‘exit’ strategy focused on a retreat from complex supply chains and interactions, towards more localised and inward facing alliances [144]. In essence signalling a retreat from political sphere debates. Thus, while some transition visions have socio-ecological benefits, they also suggest a diminished set of alliances among different groups at various scales, which brings its own limitations and concerns, not least of all towards greater populist movements which can give preference or exclude some groups in society in detriment of others. Sage suggests that given these risks iterative and reflexive processes are required to anticipate problematic practices, which seek to frame change around a ‘voice’ rather than ‘exit’ strategy and that look to maintain and reinforce crucial democratic and cooperative ties among different groups in society.

Exploring Irish media representations of climate change, Fox and Rau [139] speak of an ‘imagined public’ which is largely at odds with the practices and experiences of a broader Irish audience and which forecloses ownership and agency over climate matters by placing a strong emphasis on the consumer-citizen. This version of the ‘public’, the authors argue, reinforce more limited monetary arguments on which decisions and attitudes are framed. The authors suggest deliberative democracy processes to broaden media debate and frame citizen action within this. This research considers the way some discourses around climate change and sustainability can be alienating to some groups (namely, groups with less formal education) which actively undermines efforts towards climate action, ownership and personal interaction over certain issues. These findings overlap with those put forth by Revez et al. [142] which explores discursive dissonances in conceptions around nature and risk with impacts regarding inclusive participation in flood management and conservation policy arenas. Equally, Lennon and Scott observe tensions in the way different interest groups frame the deployment of large wind energy projects. The use of diverging scales (from national to local) to present levels of opportunity or impact, largely creates a situation where groups talk ‘past one another’ [9]. The emergence of opposition groups such as the Lakelands Windfarm Information Group (LWIG) is seen to stem from these tensions linked to major plans for proposed windfarms in the midlands, which are effectively experienced by locals as ‘sacrifice zones’ to facilitate the national transition process [123, 145].

## Conclusions

With the aid of the three spheres of transformation framework this paper has attempted to unravel emerging concepts of public engagement, drawing upon cross-sectoral and cross-disciplinary insights that have seldom been leveraged in other reviews. We expanded on the concept of emergence as a trait of public engagement, moving beyond more static perspectives that commonly bring their own prescriptive views on participation. The framework we set out offer a basis to consider multiple ‘publics’ within a transition context, seeking to capture and trace these processes as they emerge and evolve. We have highlighted the increasingly siloed and fragmented way in which public engagement in transitions is carried and we argue for a more cross-sectoral and cross-disciplinary approach which depends on bringing into dialogue often contrasting theories and perspectives. Recognizing the need to integrate what is a growing and fragmented field we use the three spheres of transformation framework to bring together different approaches to processes of change in response to concerns over climate change and environmental collapse. Seeking to overcome conceptual divides, to learn from both core and peripheral perspectives and to capture emerging conceptions of public engagement in this highly dynamic, and evolving field. The focus on emergent ‘publics’ provides a broad lens to help locate participation as a means to adapt, learn, respond or contest sustainability transition narratives, processes and outcomes [4, 5]. The value of looking across the three spheres of transformation for promoting transformative spaces of participation lies in a commitment toward reflexivity. This process is useful to make the connections often missing between the material development of technologies, structures of power, and the personal, unique and often conflicting perspectives which colour our worldviews. Attention to emergence and the interconnected ways in which concepts can evolve has provided a broader framing from which to explore public engagement dynamics. The nested view that the three spheres of transformation proposes encourages us to explore across different layers of action and debate, namely, the practical, political and personal spheres. Furthermore, it also encourages us to transcend myopic paradigms to unravel further opportunities for transformation and change [24, 146].

Focusing on the Irish context, the findings derive from literature from the last 20 years. They show that this is a relatively recent field of research and policy development. The findings show that there is a wealth of ideas and proposed pathways which place and define the ‘public’ in quite diverse ways. While there is an abundance of articles addressing the political and practical spheres of transformation, there is somewhat of a dearth of articles dealing with the personal sphere. This suggests a gap in accounting for the influence of values and worldviews in the configuration of the systems and structures we are attempting to disrupt, transform and transition. Equally, the scoping review found very few articles tackling sustainable transitions from a cross-sectoral perspective and those that we found consisted mainly of grey literature. This may suggest difficulties in reconciling complex cross-sectoral insights within more prescriptive theoretical structures. The integrated analysis of three spheres approach also allows us to consider different contexts. We have identified some shifting engagement approaches. For instance, the nexus articles between the practical sphere and the political sphere propose deeper forms of public engagement beyond aggregated consumer behaviour to align technological delivery with institutional and societal contexts. While most articles in the practical sphere draw largely on techno-economic insights this influence and cross-disciplinarity is likely to draw in further innovations. Nexus articles between the political and personal sphere are also drawing on shifting ideas of public engagement and largely stress the need to disrupt pre-conceived notions of engagement and agency within our institutions. These articles highlight the problem with top-down public engagement structures and in various ways show how they undermine and marginalise different groups.

A number of emerging public engagement processes stand out as important public formations. The increased use, and public acceptance, of Citizens’ Assemblies within the Irish policy landscape has created space for showcasing innovations, leadership and good practice and for the sharing of personal testimony. In addition, their focus on informed deliberations, emphasises the role of scientific evidence in policy making as well as the creation of new arenas for researchers to share their findings in an open, accessible way and where they are questioned, clarified and considered.

Yet, they are not a panacea for all that ails governance processes for a couple of reasons. First, they are still essentially ‘top down’ processes as they are, within an Irish context, dependent on (a very centralised) structure of Government for their establishment, resourcing and legislative impact. Second, they are but one part of a wider democratic system which includes parliament, the free press, wider civil society, activists, etc. Certainly there is a body of research that shows that they can, if well designed and properly resourced, provide deliberative moments within this system. However, deliberation, in terms of informed, other regarding, inclusive and respectful argument, cannot and should not be solely confined to Citizens’ Assemblies [147, 148] that are formed on an ad hoc basis and that are potentially open to co-option and the ‘cherry picking’ of their proposals [149].

Community ownership is another dominant idea in the literature with applicability across of range of different contexts from the creation of microenergy generation and food cooperatives, localisation movements (for example the Transition Town movement), the development of local social enterprise models, to concepts such as citizen investors and prosumers. By and large these can be a way to ease local tensions, foster social acceptance of new technologies, broaden the investment base and increase community engagement and democratic processes in sustainability transitions. Yet, there are critical and unresolved issues tied to these ideas. Not least of all that these remain aspirational visions with limitations in terms of direction and insights towards distributed and community ownership models [100]. Other concerns include the fact that ‘ownership’ may lead to a commodification and marketisation of societal needs and priorities by giving undue focus to financial discourses while neglecting to resolve key political questions [101]. Assumptions about local networks as inherently democratic and made-up of ‘convivial communities’ [13] are also potentially problematic and may lead to unforeseen processes of exclusion and marginalisation. Ultimately, the concern is that the concept of community ownership is used as a proxy for adequate political participation leading to exclusion rather than participation.

Finally, opposition groups emerge as important forms of engagement in sustainability transitions, not just from expected protest the siting of new energy technologies but also in response to approaches adopted in water policy, divestment from carbon, and flood management strategies. The role of opposition groups for system disruption and regime change remain highly relevant. In particular, because they offer the most significant form of organised resistance with regard the physical and environmental costs of sustaining an ever expanding neoliberal industrial system [120]. This disruptive capacity can also be a barrier toward deeper and accelerated processes of transition, when environmental issues become conflated with other concerns as was the case of the Right2Water campaign. They also outline the importance of acknowledging public values in the promotion of new policy and the agency of communities to counter these policies, when they are perceived to undermine these values. As with other forms of participation, protest and activism interventions are best located within a wider space among other engagement and participation fora to shape and frame a more meaningful public engagement arena.

We acknowledge limitations in this scoping process, while we strived to provide a comprehensive overview of existing research, we do not claim to offer a complete map of all available literature. Equally we acknowledge limitations in translating the findings of our research into more generalisable conclusions. Nevertheless, the exercise carried in mapping public engagement processes in Ireland demonstrates the need for more reflexive and cross-disciplinary frameworks, which we hope will inspire new and broader research questions. Additional comparative research beyond the Irish context would doubtlessly provide valuable insights to either validate or contest our findings within different settings. Taking stock of the literature on transitions and public engagement at different points in time is particularly useful to help make sense of a continuously shifting space of emerging groups and interventions. Adopting a more pragmatic approach using the three spheres of transformation has enabled us to draw in cross-disciplinary and cross-sectoral perspectives which is seldom considered in other literature. In our view, adopting this wider perspective is a valuable opportunity for enriching the public engagement debate either building on some of the ideas we outline, contributing with further insights or indeed contesting the approach.

## Data Availability

Not applicable.

## Abbreviations

ASC: Academic Search Complete
ASSIA: Applied Social Sciences Index and Abstracts
cVPP: Community-based Virtual Power Plants
EPA: Environmental Protection Agency
EU: European Union
EV: Electric Vehicles
GHG: Green House Gas
GW: Gigawatt
HEI: Higher Education Institution
ICT: Information and Communication Technologies
IPCC: Intergovernmental Panel on Climate Change
MLP: Multi-Level Perspective
OPW: Office of Public Works
PPN: Public Participation Networks
PV: Photovoltaic
SDG: Sustainable Development Goals
SEAI: Sustainable Energy Authority of Ireland
TM: Transition Movement

## Footnotes

1 The OPW is the lead state-agency charged with the national flood risk management plans in Ireland.
